# Calculation of Fetal Fraction for Non-Invasive Prenatal Testing

**DOI:** 10.3390/biotech10030017

**Published:** 2021-08-09

**Authors:** Matthew Cserhati

**Affiliations:** Independent Researcher, 2615C Muscatel Ave., Rosemead, CA 91770, USA; csmatyi@protonmail.com; Tel.: +1-531-301-1167

**Keywords:** fetal fraction, NIPT, cfDNA, aneuploidy, differential methylation, SNP quantification, length distribution estimation, Y chromosome, SeqFF

## Abstract

Estimating the fetal fraction of DNA in a pregnant mother’s blood is a risk-free, non-invasive way of predicting fetal aneuploidy. It is a rapidly developing field of study, offering researchers a plethora of different complementary methods. Such methods include examining the differences in methylation profiles between the fetus and the mother. Others include calculating the average allele frequency based on the difference in genotype of a number of single-nucleotide polymorphisms. Differences in the length distribution of DNA fragments between the mother and the fetus as well as measuring the proportion of DNA reads mapping to the Y chromosome also constitute fetal fraction estimation methods. The advantages and disadvantages of each of these main method types are discussed. Moreover, several well-known fetal fraction estimation methods, such as SeqFF, are described and compared with other methods. These methods are amenable to not only the estimation of fetal fraction but also paternity, cancer, and transplantation monitoring studies. NIPT is safe, and should aneuploidy be detected, this information can help parents prepare mentally and emotionally for the birth of a special needs child.

## 1. Introduction

Screening for fetal aneuploidy or other fetal genetic defects are an important part of prenatal testing. Until relatively recently, genetic testing of the fetus was done via amniotic fluid testing and chorionic villus sampling (CVS). However, these tests have a risk of infection and also miscarriages [1,2], with rates of around 0.1–0.3% and 0.5%, respectively [3].

In 1997, it was discovered that a small fraction of the fetus’s DNA can be detected in the mother’s blood, besides the mother’s DNA after seven weeks of gestation [4]. Together, these make up cell-free DNA (cfDNA). The placenta sheds cells, and with it, cell-free fetal DNA (cffDNA), which is made up of fragments usually 50–300 bp long. The fraction of all DNA circulating in the mother’s blood that originates from the fetus is called the fetal fraction (FF), expressed as a percentage value.

Researchers can exploit the differences between the fetal DNA and the mother’s DNA to estimate FF. Estimating FF is crucial in testing for chromosomal abnormalities, such as trisomies 13, 18, and 21 (T13/18/21), (the most common) or monosomy X [5] (see Table 1).

Since chromosome 13 is larger than chromosomes 18 and 21, an extra copy of chromosome 13 will increase FF. Trisomy 13 is also rarer than trisomy 18, which in turn is rarer than trisomy 21, because the number of genes affected by these abnormalities is proportionate to the size of the chromosome. Clinical results have also shown that estimating the FF is quite effective in predicting fetal aneuploidy [6]. It is preferable over amniocentesis or CVS, since due to its non-invasive nature, it only requires drawing blood from the mother, excluding the risk of miscarriage. By 2007, the first non-invasive prenatal tests were devised, based on the measurement of FF.

The average FF may vary from study to study, but is generally between 10% and 20% of all blood plasma [7,8], but can range from less than 4% to more than 30% [9]. A minimal FF of 4% is usually necessary to be able to detect fetal aneuploidy or other genetic defects. Around 2% of tests showed an FF of less than 4%, and 2–6% of NIPT samples are sent back for redrawing at late GA due to low FF [10]. Intuitively, a higher FF value indicates a higher chance of fetal aneuploidy [11,12]. Thus, an FF of 4% from a trisomy 21 fetus corresponds to a 2% increase in the proportion of DNA fragments from chromosome 21.

In what follows, the factors influencing FF, and different methods of detecting FF are discussed.

## 2. Factors Influencing Fetal Fraction

### 2.1. Maternal Factors Influencing Fetal Fraction

There are several maternal factors that influence the detectable FF in the mother’s blood, such as gestational age (GA) and maternal weight (MW). Interestingly, maternal age, though an indication of higher risk for fetal aneuploidy, does not influence FF significantly. The longer the mother has been in gestation, the more the time that has passed, allowing for cffDNA to accumulate in the mother’s blood. Thus, there is a statistically significant positive correlation between gestational age and FF [13]. Conversely, the greater the maternal weight, the lower the FF. This is because with a larger body mass index (BMI), the mother will shed more maternal DNA into the blood stream, in effect decreasing the proportion of cffDNA. Dar et al. showed a significant negative correlation between FF and MW [13].

Confined placental mosaicism (CPM) might also give the impression of a false positive if the placenta is aneuploid but the fetus itself is not. The fetus or the placenta could be mosaic. If only the fetus contains cells containing aneuploid DNA, this is a false negative (FN); that is, if the aneuploid fetus goes undetected [3].

Differential methylation of certain genes or other segments of the genome can also help differentiate between fetal and maternal DNA. Several NIPT technologies make use of this fact to determine the FF.

A vanishing or unreported twin may produce a false-positive (FP) result, since the extra DNA coming from the vanished twin gives the impression of fetal aneuploidy (extra chromosomes). Vanishing twins make up to a third of all FP cases of fetal aneuploidy [14]. This is all the more significant, since the ratio of twin births has doubled between 1980 and 2009 to 1 out of every 30 births [15]. This proportion rises to 9% of conceptions, resulting in vanishing twins through intracytoplasmic sperm injection [16]. Higher MA also increases the likelihood of vanishing twins, and the vanishing twin can contribute cffDNA for up to eight weeks after demise [17]. In the case of twins, the FF is higher compared to a singleton pregnancy. However, the total FF is less than what would be contributed by two individual twins [18].

Conversely, the presence of aneuploidy in human gametes and embryos is a major cause of failure in in vitro fertilization (IVF) and miscarriages. Comparative hybridization techniques and NGS techniques have been developed that are able to assess all chromosomes, not just chromosomes 13, 18, and 21 for preimplantation genetic testing (PGT) during IVF. A drawback of IVF-mediated pregnancies is that due to the nature of the technology, the FF may appear to be lower due to variation in placental serum markers, such as pregnancy-associated plasma protein-A (PAPP-A) and beta-human chorionic gonadotropin (β-hCG) [19,20]. This is because these two factors promote the development of the placenta, thereby increasing the FF. However, according to Lee et al. [21], inflammation might disrupt maternal endothelial cells, thereby increasing maternal cffDNA relative to the amount of fetal cffDNA. Even the process of freezing and thawing embryos reduces the level of PAPP-A during IVF [22].

A study by Lee et al. [23] of 5625 singleton pregnancies showed that IVF-induced pregnancies had a median FF of 10.3% as opposed to a median value of 11.9% in spontaneous pregnancies, across MA, GA, BMI, and ethnicity. This increases the false-negative rate of detecting aneuplodies in IVF-mediated pregnancies [24]. In the same study by Lee et al., PPV declined from 73.4% in spontaneous pregnancies to 28.6% in IVF-induced pregnancies.

Other factors, listed in Table 2, may influence or falsify the FF. Because of these factors, FF-based aneuploidy testing does not provide a conclusive diagnosis. Conversely, a negative result does not signify an unaffected pregnancy [25].

### 2.2. Non-Fetal Factors as Causes for False-Positive NIPT Results

Besides FF, several non-maternal factors may falsely suggest the appearance of aneuploid fetal DNA in the mother’s blood.

Maternal neoplasms such as leukemia, lymphomas, breast cancer, colorectal cancer, myelomas, and uterine fibroids might also provide false positives. In these cases, the FF measurement may be analytically correct, but not clinically correct. This is because cancerous cells also undergo aneuploidy and enter the circulation [26,27]. The most common types of gestation-associated malignancies include breast cancer, cervical cancer, Hodgkin’s disease, melanoma, leukemia, ovarian cancer, and colorectal cancer [28,29]. Table 3 shows the incidence of the leading causes of maternal cancers during pregnancies [30]. However, tracking down the exact cause of the malignancies in such false-positive NIPT is difficult, especially since NIPT is geared more towards aneuploidy of chromosomes 13, 18, and 21. The rate of diagnosing a pregnancy-associated malignancy during NIPT is between 1 for every 1000 or 2000 20- to 40-year-old women [30]. Furthermore, a diagnosis of multiple aneuploidies carries a 20–44% risk of maternal cancer according to a study by Bianchi et al. [31].

Furthermore, even tissue from male donors or blood transfusions from a male may falsify testing for fetal genetic defects due to the possible presence of Y chromosome material from the transplanted tissue in the mother’s blood. This could give the impression that a female fetus’s gender is really male [32,33].

These confounding factors may require a repeat blood draw, especially if the GA is too low and the MW is too high. A repeat blood draw might produce better results after waiting several weeks for more cffDNA to accumulate. Wang et al. [34] report that more than 99% of pregnant woman under 70 kg had an FF of ≥4%. However, only 71.4% of pregnant women under 90 kg yielded an FF of ≥4% on the second draw.

## 3. How to Measure Fetal Fraction

Four main methodologies have been described in the NIPT literature using different approaches to estimate FF. All of these methods make use of different aspects of next-generation sequencing (NGS) technologies to help in the estimation. These include differential methylation methods, quantification of single-nucleotide polymorphisms (SNP), Y chromosome-based DNA fragment estimation, machine learning algorithms, and fragment length distribution estimation methods [35]. A tabular overview of these methods can be seen in Table 4 with their advantages and disadvantages [36].

### 3.1. Differential Methylation Methods

Genomic methylation patterns change over time, so therefore it is no surprise that the methylation state of the fetal genome is different than that of the mother. Bisulfite sequencing has been used to discover differentially methylated genome regions between the placenta and all other tissues [37]. There are 16 regions of the genome which are known to be differentially methylated between the fetus and maternal blood cells. Hypomethylated regions tend to have both low GC% and low gene density. However, since only 20–30% of CG islands in the human genome are unmethylated [38], their targeted analysis could make NIPT more cost-effective. Differential methylation technologies consume less genomic material than do the SNP-based method (see later). This also recues the cost of methylation NIPT methods.

Twelve of these hypomethylated regions are located on chromosome 12, and four on other chromosomes [39]. For example, the phosphodiesterase gene, PDE9A on 21q22.3 is completely methylated in maternal blood cells, but unmethylated in the placenta. Another common methylation marker gene is SERPIN5B, located on chromosome 18, which is also hypomethylated in the placenta but not in maternal blood cells [40]. The ratio of SNP between in the hypomethylated version of SERPIN5B might help detect T18 [41]. Conversely, the promoter of the RASS1FA gene is hypermethylated in the placenta, whereas it is hypomethylated in maternal blood cells [42]. The presence or absence of these marker genes can be detected by methylation-sensitive restriction enzymes followed by quantification by real-time polymerase chain reaction (RT-PCR).

For the detection of differentially methylated DNA fragments, two next-generation sequencing methods are amenable, namely uTOP-seq and hmTOP-seq [43,44]. These two methods are capable of determining unmodified CG-dinucleotides (uCG) as well as 5-hydroxymethylated cytosines in CG-dinucleotides (5hmCG). The signal strength of these two methods is higher than that of non-pregnant controls (NPC). The FF increases proportionately to the increasing read counts found by uTOP-seq. Conversely, the FF decreases according to the number of reads found by hmTOP-seq [45].

The fetal quantity assay (FQA) by Nygren et al. [46] uses restriction enzymes to digest the unmethylated maternal DNA, leaving the hypermethylated genomic region including the two marker genes SOX14 and TBX3 in the cffDNA intact. Their method involves mixing in a competitive allele together with the target DNA, which is identical in sequence except for a single bp mismatch. The competitive allele was designed in such a way that it was heavier than the target and could be separated using matrix-assisted laser desorption/ionization time-of-flight mass spectrometry (MALDI-TOF MS). Since the quantity of the competitive allele is known, as well as the ratio of the target and competitive allele, the proportion of the target DNA can be deduced, thus yielding the FF.

### 3.2. Quantification of SNPs

SNP quantification methods involve measuring the presence of reads containing single base pair mutations from the fetus. Two main factors are the most important in calculating the FF in these methods: read depth [47] and the number of SNPs used in the analysis [48]. The deeper the genome coverage and the more the SNPs used in the analysis, the better the results. However, there is a trade-off between coverage and the number of SNPs on one hand and cost on the other. The SNPs must also be common enough in the general population to be detectable so that they can be used. SNPs can be selected from various online databases, such as HapMap, gnomAD, or dbSNP [49].

Whole genome-wide (WG) studies are the most accurate, since they detect genetic anomalies throughout the entire genome, as opposed to localized positions within the genome. However, as opposed to localized targeted studies, the cost for performing WG studies is higher, especially if both parents need to be genotyped. WG studies are also not limited by the fetus’s gender as in Y chromosome studies [50].

When interpreting results, care must be taken with interpreting the minor allele frequency (MAF), otherwise known as the alternate allele or variant allele. With lower MAFs, the chance of error increases [51]. Non-maternal alleles may come from the fetus, but they may be due to sequencing and genotyping errors [48]. Table 5 describes the different classes of minor alleles based on frequency [52,53].

Now the question is, how can we calculate FF in practice using SNP data? One way is to extract DNA from the mother’s blood and generate NGS reads. The reads can then be aligned to a version of the human genome (usually hg19 or hg38) using an aligner program, such as bowtie, bwa, or SOAP2. After alignment a variant calling program (such as VarScan2, TNscope, or MuTect) can then be used to call variants. The variant caller must call somatic variants as opposed to germline variants, and the genotype also has to be known.

A method developed by Zhang et al. [54] calculates the mean maternal and paternal variant allele fraction (VAF_m_, VAF_p_) in order to calculate FF. If a variant is present, it could be due to either one of two cases. The mother could have the homozygous reference allele (A), whereas the fetus inherited the variant allele (B) from its father. In such a case, the paternal variant allele fraction (VAF_p_) should be proportionate to half of the FF. Or, the mother is homozygous for the alternate allele (B), whereas the fetus is heterozygous (AB), inheriting the reference allele from its father. The maternal variant allele fraction (VAF_m_) is thus 1-FF/2. Rearranging the equations, and calculating FF over all selected alleles in the study, the FF according to Zhang et al. is:

(1) $$\mathrm{FF}=1-\mathrm{mean}\left({\mathrm{VAF}}_{m}\right)\;+\;\mathrm{mean}\left({\mathrm{VAF}}_{p}\right)$$

**Figure 1:**
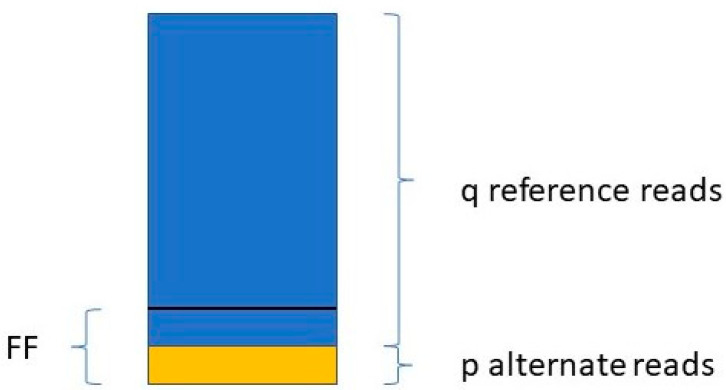
Calculating fetal fraction from a homozygous. mother and heterozygous fetus regarding a single SNP.

Jiang et al. [48] determine the FF in a similar way. They take only those variants into account where the mother is the homozygous reference (AA) and the fetus is heterozygous (AB). For a given variant, if the number of reads with the reference allele is q, and the number of reads with the variant allele is p (see Figure 1),

then:

(2) $${\mathrm{FF}}_{i}=\frac{2p}{p+q}$$

The FF is equal to the average of all FF_i_ values, where i ranges from 1 to the number of SNPs included in the study.

### 3.3. Read Length and Read Count Distribution Methods

Other technologies make use of the fact that as fetal DNA is shed into the mother’s bloodstream, the DNA molecules get fragmented decreasing in length. Similar technologies make use of the fact that in the case of chromosomal or regional aneuploidies, more (or less) reads map to that chromosome or region than on average. However, read coverage is influenced by gene density and exon count [55]. In these cases, the genome can be divided into bins (usually 50–100 Kbp but even 1 Mbp), and subregions of the genome can be compared to the median according to the number of reads that map to it [56]. This is the principle behind the software SeqFF, which is widely used in FF estimation [55]. Here the ratio of fragments less than 150 bp compared to fragments less than 600 bp long is measured in 50 Kbp bins across the genome.

Fetal DNA fragments are on average shorter than maternal DNA fragments [57]. As the FF increases, shorter DNA fragments increase in frequency. In fetal trisomy 21, the proportion of shorter reads would increase due to the extra chromosome copy. Conversely, in monosomy X the proportion of longer fragments of maternal origin increases. So, for example, we know that chromosome 21 makes up 1.56% of the entire human genome (46.7 Mb/3 Gb). If we find that the DNA fragments coming from chromosome 21 in the blood sample are significantly larger than 1.56%, then we can infer fetal aneuploidy. Since we also know that in T21 chromosome 21 is present in three copies, the cffDNA should contribute [1.5 × 1.56 FF]% of reads mapping to chromosome 21.

Therefore, technologies which exploit the differences in length between fetal and maternal DNA fragments mainly use paired-end reads, instead of single-end reads. Short single-end reads accumulate all across the genome. However, single-end reads can still be used in other ways. Paired-end reads, on the other hand, can be used to deduce cfDNA fragment lengths because of their insert size.

**Figure 2:**
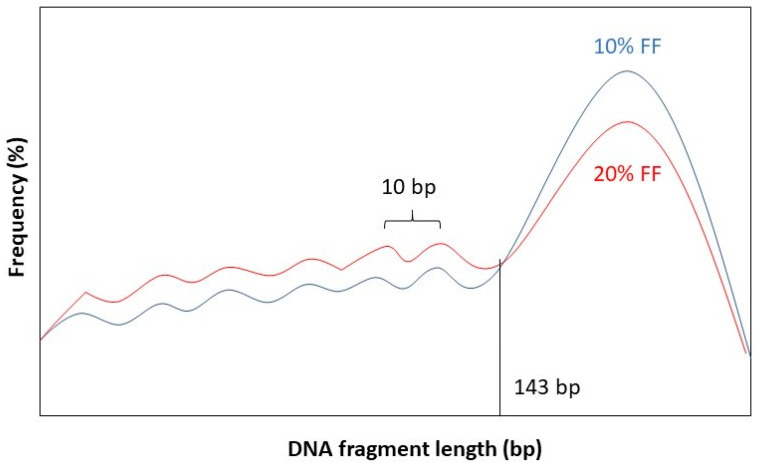
Length distribution of fetal DNA fragments according to different values of the FF: 10% (blue) and 20% (red).

It has been observed that the fragment length boundary between short and long DNA fragments corresponds to 143 bp. This is exactly the length of a DNA molecule that wraps around a histone protein. Below 143 bp DNA fragment lengths show peaks with a periodicity of 10 bp. This 10 bp periodicity corresponds to a full 360° turn in the DNA helix, after which nuclease-sensitive sites are exposed on the DNA’s surface [58]. Furthermore, while the general shape of the distribution stays the same for different values of FF, the amplitude of the distribution curve varies. See Figure 2 for a graphical representation.

Dheedene et al. [59] found a tight correlation between the FF and the z-score calculated from the distribution of DNA fragments mapped to each chromosome. A distribution shifted towards shorter DNA fragments indicates a higher FF, and vice versa. The authors were able to achieve a 98%, 94% and 100% sensitivity for T21, T18 and T13, respectively.

The concrete amount of cfDNA fragments within a given size interval can be also estimated by examining the area under the curve (AUC) of a bioanalyzer electropherogram [60]. A fragment length ratio can be deduced by dividing the two AUC values for fragments between 78 and 143 bp long and fragments between 163 and 168 bp long. This procedure can be done in the case of T13/18/21, where the proportion of short fragments can be compared for the affected chromosome (i.e., chromosome 21) and all the other autosomes minus chromosomes 13, 18 and 21. A z-score can be calculated by subtracting the autosomal short fragment proportion from the long fragment proportion. A z-score with an absolute value greater than 3 is indicative of fetal aneuploidy [60].

### 3.4. Y Chromosome-Based Methods

Another class of methods make use of the fact that a male fetus carries DNA which the mother to large extent does not have: the Y chromosome. This means that the number of DNA fragments matching the sequence of the Y chromosome should be directly proportionate to the FF. However, this is not so straightforward, since a significant portion of the Y chromosome, called the pseudo-autosomal region (PAR), is 98% homologous to the q13–q22 region of the X chromosome [61]. The PAR even recombines during meiosis [62].

**Figure 3:**
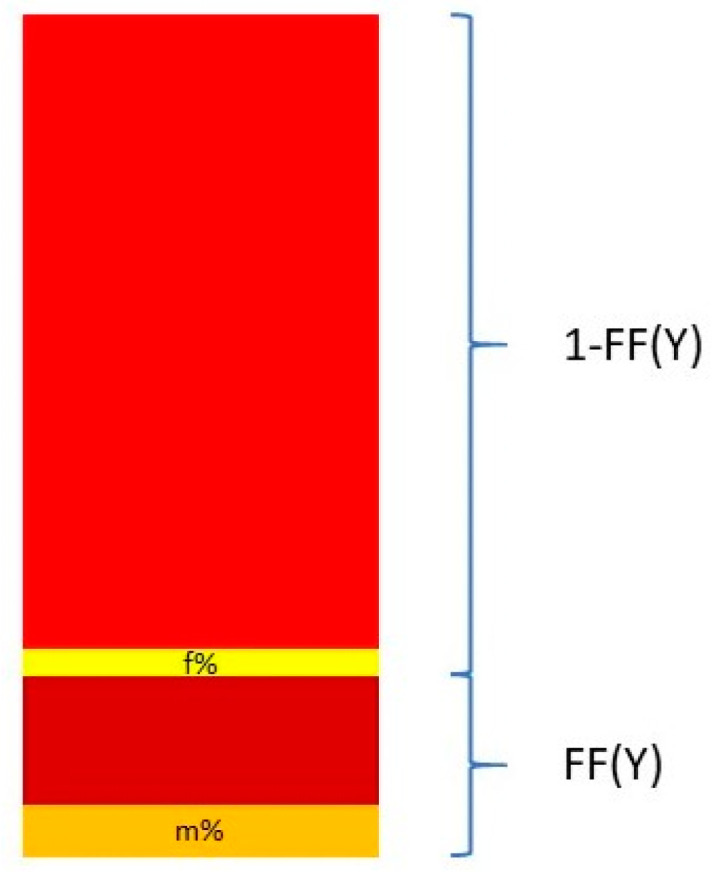
Calculation of the FF based on proportion of Y chromosome reads mapping to the Y chromosome and the pseudo-autosomal region of the X chromosome.

Nevertheless, Y chromosome-based FF estimation is fairly accurate. The two drawbacks are that only male fetuses can be tested and that the small size of the Y chromosome can lead to high variation in measurements [63]. This means that a small portion DNA fragments from a cfDNA sample that map to the Y chromosome come from the PAR of the X chromosome. This is described in Figure 3.

The relationship between the FF and the proportion of reads coming from the Y chromosome and the PAR of the X chromosome can be described in the following equation:

(3) $$Y\%=Y_{\mathrm{male}}\%\cdot \mathrm{FF}\;+{\;Y}_{\mathrm{female}}\%\cdot \left(1\;-\;\mathrm{FF}\right)$$

and thus

(4) $$\mathrm{FF}=\frac{Y\%\;-{\;Y}_{\mathrm{female}}\%}{Y_{\mathrm{male}}\%\;-{\;Y}_{\mathrm{female}}\%}$$

where Y% is the total percent of reads that map to the Y chromosome. Y_male_% and Y_female_% are the proportion of the Y chromosome reads mapping to the Y chromosome and the PAR of the X chromosome, respectively. These two quantities are measured from a control set of adult males and pregnant women bearing euploid female fetuses [64,65].

Mazloom et al. [63] developed a method for detecting sex chromosome aneuploidies (SCA), such as Turner syndrome noted as [45, X], trisomy X [47, XXX], Klinefelter syndrome [47, XXX], and Jacobs syndrome [47, XYY]. About half of all fetal aneuploidies involve SCA, affecting 0.3% of all live births. In their algorithm, the number of normalized reads matching a region corresponding to 76.7% of the X chromosome and 2.2% of the Y chromosome was determined and compared to autosomal read counts. This proportion was then compared to ratios derived from control samples. These control samples were derived from a set of 480 pregnancies with euploid female fetuses for the X chromosome, and 23 pooled male adults for the Y chromosome.

## 4. Software for Estimating FF

### 4.1. Gold Standard

There exists a gold standard in NIPT whereby the FF can be measured. Wald et al. [35] studied T21 in a set of 62 singleton affected pregnancies and 3785 unaffected pregnancies. According to their calculations, the percentage of DNA fragments from a pregnancy affected with T21 (P_21_) is proportionate to

(5) $$P_{21}=P_{U}\left(1-\mathrm{FF}\right)+\left(\frac{3}{2}\right)\cdot P_{U}\cdot \mathrm{FF}=\left(\frac{1}{2}\right)\cdot P_{U}\cdot \mathrm{FF}+P_{U}$$

where FF is the fetal fraction, and P_U_ corresponds to the proportion of DNA fragments from chromosome 21 in an unaffected pregnancy. According to Wald et al.’s estimates, P_U_ = 1.2935%. Since the proportion of DNA fragments coming from chromosome 21 can be measured, the FF can be inferred from this equation. Based on known FF values, Wald et al. [35] compared the results from SeqFF when run-on affected pregnancies and found an extremely tight correlation between the gold standard FF values and the ones estimated by SeqFF. Wald et al.’s model is useful because the accuracy of any kind of FF determining algorithm or method can be measured by it.

### 4.2. FF Software

Besides the work of Wald et al., a number of different software have been developed to estimate FF based on one or even a combination of the previously detailed technologies. A list of several well-known FF estimation software can be seen in Table 6.

One of the older software that was used early on after NIPT became a technology is Next-Generation Aneuploidy Test Using SNPs (NATUS). This software uses parental and fetal genotype data, known inheritance patterns, and targets 19,488 SNPs. It uses a complex Bayesian maximum likelihood algorithm to determine the likelihood of each ploidy hypothesis (trisomy, disomy, and monosomy) and FF value given the data [66]. Newer technologies are capable of determining the FF using only several hundred SNPs. SNP testing is also capable of detecting the presence of dizygotic twins from multiple haplotypes. The NATUS algorithm does this by detecting differences between the predicted most likely fetal haplotype and the observed fetal allele distributions [17]. This method cannot detect monozygotic twins, however.

The software SeqFF [55] is the most common method used to determine the FF in NIPT studies. The basic principle involves discovering read overrepresentation in sub-chromosomal regions of 50 kbp. The method ignores information from sex chromosomes and is thus applicable to both male and female fetuses.

Jiang et al. [48] devised an algorithm called FetalQuant^SD^ and found a linear relationship between the ratio of non-maternal alleles and FF in a maternal blood sample. According to their equation,

(6) FF=18.9X − 6.6

where X stands for the non-maternal allele fraction (the total number of non-maternal alleles divided by the total number of alleles times 100). They also showed that the FF estimate is more accurate if more SNPs are involved in the study and the higher the number of reads.

DEFRAG is a method that detects the FF in male fetuses. It does so by measuring the proportion of reads mapped to the Y chromosome in a pregnancy with a male fetus by comparing it to two baseline measurements of 0 and 100% male DNA [67]. These are denoted as %Y_XX fetus_ and %Y_XY man_, respectively. If %Y_XY fetus_ denotes the percentage of reads mapped to the Y chromosome in a male pregnancy, then

(7) $$\mathrm{FF}=\frac{{\%Y}_{\mathrm{XY}\;\mathrm{fetus}}-{\;\%Y}_{\mathrm{XX}\;\mathrm{fetus}}}{{\%Y}_{\mathrm{XY}\;\mathrm{man}}}$$

BAYINDIR measures the median read coverage over all autosomes in 50 kbp bins and compares it to median read coverage over the X chromosome, as well as those 50 kbp segments of the Y chromosome, which are dissimilar to the X chromosome [68]. Large deviations indicate SCA.

SANEFALCON makes use of the difference in DNA fragment length between the mother and the fetus [69]. Fetal DNA is cut at the linker sites between nucleosomes, increasing the number of short DNA fragments. Compared to the maternal DNA where the DNA linker is intact; thus, the maternal DNA should be made of longer DNA fragments.

Comparing these methods with one another, DEFRAG may be used the most efficiently to detect aneuploidy in male fetuses, whereas seqFF may be used to detect aneuploidy the mist effectively in female fetuses. FF estimates by SANEFALCON gave moderate correlations with DEFRAG and SeqFF [70].

Miceikaitė et al. [71] studied FF as a function of the number of analyzed reads, using SeqFF. They found that FF varied greatly when using less than 10 million reads for each sample. The authors recommend using at least this many reads in FF estimate studies. However, the accuracy of FF estimates by the SeqFF software were stable across a range of FF values between 2–13%. Accuracy did not improve much over an FF of 20%.

The goal of any FF estimation software is to correctly detect fetal aneuploidy. The most optimal would be to keep the false-positive (FP) rate and false-negative (FN) rates as low as possible. Remember that FPs and FNs arise when the result is analytically correct, but clinically incorrect. FPs arise in such cases when the placenta may be aneuploid but the fetus is not, or in cases of vanishing twin. FNs arise when the fetus is really aneuploid, but this fact goes unreported, as when the placenta is unaffected but the fetus is aneuploid. FF estimates should have a high sensitivity, a high specificity, and high precision. These statistical measures are defined in Table 7.

## 5. Discussion

### 5.1. Summary of FF Estimation

Estimating FF is a rapidly developing field. Much progress has been made over the past two decades in order to make the estimation of FF quicker and more accurate. Researchers and clinicians have a plethora of options to choose from. A combination of these methods can be used to complement one another to accurately estimate FF.

The analysis of FF is amenable not only towards diagnosing fetal aneuploidy. The basic principle of detecting DNA present in small quantities can be used in other technologies as well. For example, SNP analysis can be used in paternity testing, both prenatally and postnatally. In such studies, fetal short tandem repeats from the Y chromosome (Y-STRs) may be used besides SNPs. The drawback of using Y-STRs is that relationships cannot be excluded within the same male lineage, and the high mutation rate of Y-STRS increases the number of false negatives [72].

Cancer diagnosis is another area of research where detection of low quantities of DNA can be made use of. Existing NIPT technologies can be slightly adapted to discover maternal cancers [73]. Chan et al. [74] showed that the size of circulating cfDNA in nasopharyngeal cancer patients was significantly larger than in control patients. For another example, a small number of cancer cells may remain in the bone marrow after treatment. This condition is known as minimal residual disease (MRD). Malignant B cells and T cells contain very specific clonal arrangements of immunoglobin (IG) genes and T cell receptors. NGS-based technologies could be used to detect the presence of these lymphocytes [75].

A similar phenomenon occurs with organ transplants. During the transplantation process, the transplant cells undergo apoptosis and empty their contents into the recipient’s blood stream. As such, the DNA gets fragmented, and this lends itself to fragment length difference analysis as described in a previous section [76].

### 5.2. Ethical Considerations

Calculating FF can help determine aneuploidy or other genetic defects in the fetus. A positive diagnosis is information that must be taken with great care. In no ways must this information be used to make the decision to terminate the pregnancy. Rather, this information can help the parents prepare for a special needs child. Kaposy argues that social practices based on biases against cognitive disabilities is objectionable [77]. According to a study by How et al. [78], some fathers of mentally handicapped children give voice to their fear that NIPT could lead to eugenics, and that many of those who did not terminate their Down syndrome pregnancy even described their mentally handicapped child as a source of joy in their lives.

According to an opinion poll, 96% of Ph.D. biologists, regardless of their worldview think that life begins at conception [79]. In the Bible, Jeremiah 1:5 amazingly says: “Before I formed you in the womb I knew you; before you were born I sanctified you.” According to Bunnik et al. [80], “on a personal level, for instance, it is perfectly possible to love and cherish a child with a disability.” Indeed, each life is precious, and each life is worthwhile.

## Figures and Tables

**Table 1:** Common chromosomal abnormalities.

Name	Illness
Trisomy 13	Patau syndrome
Trisomy 18	Edwards syndrome
Trisomy 21	Down syndrome
Monosomy X	Turner syndrome
Trisomy X	Triple X syndrome
47 [XXY]	Klinefelter syndrome
47 [XYY]	Jacobs syndrome

**Table 2:** Factors influencing the FF.

Factor	Implication
Confined fetal aneuploidy	False negative; fetus appears normal when really affected
Differential methylation	Helps tell difference between maternal and fetal DNA
Gestational age	Increases fetal fraction
IVF-induced pregnancy	Decreases fetal fraction
Male blood/tissue donor	Falsification of fetal sex
Maternal cancer	Apparently higher FF
Mosaic placenta	False positive; fetus appears to be affected
Mother’s weight	Lowers fetal fraction
Vanishing/unreported twin	False positive; extra twin’s DNA gives appearance of fetal aneuploidy

**Table 3:** Incidence of leading types of maternal cancer during pregnancy (data from Pavlidis, 2002).

Type of Cancer	Incidence
Breast cancer	1:3000–10,000
Cervical cancer	1.2:10,000
Hodgkin’s disease	1:1000–6000
Malignant melanoma	2.6:1000
Leukemia	1:75,000–100,000
Ovarian cancer	1:10,000–100,000
Colorectal cancer	1:13,000

**Table 4:** Overview of different FF quantification methods.

Method	Advantages	Disadvantages
Methylation differences	Accurate	Enzymes may affect accuracy, genome-wide analysis expensive
SNP quantification	Accurate	Cost of genotyping, consumes large quantity of genomic material
Length distribution	Easy to perform	Inaccurate, but can be increased with paired-end reads
Y chromosome	Accurate and simple	Can only test male children

**Table 5:** Different classes of minor alleles based on frequency.

Type	Frequency
Common allele	5% < MAF
Low-frequency variant	0.5% < MAF ≤ 5%
Rare variant	MAF ≤ 0.5%

**Table 6:** Advantages and disadvantages of several well-known FF tools.

Software	Advantage	Disadvantage
BAYINDIR	Can identify low FF	Y chromosome-specific
DEFRAG	Can identify low FF	Y chromosome-specific
FetalQuant^SD^	No parental genotype needed	Needs large number of SNPs
NIPTmer	Fast	Does not handle extreme FF values
SANEFALCON	Can identify low FF	Non-uniform genome coverage
SeqFF	Gold standard, good for both genders	Many false positives
WisecondorX	Can use single-end and low coverage data	Exclusive for NIPT

**Table 7:** Common statistical measures used in FF estimation.

Statistical Measure	Formula	Synonym
Sensitivity	$\frac{\mathrm{TP}}{\mathrm{TP}+\mathrm{FN}}$	Recall
Specificity	$\frac{\mathrm{TN}}{\mathrm{TN}+\mathrm{FP}}$	
Positive prediction value (PPV)	$\frac{\mathrm{TP}}{\mathrm{TP}+\mathrm{FP}}$	Precision

## Data Availability

This study did not report any data.

## Abbreviations

5hmCG	5-hydroxymethylated CG dinucleotide
β-hCG	beta-human chorionic gonadotropin
BMI	body mass index
cffDNA	cell-free fetal DNA
CPM	confined placental mosaicism
CVS	chorionic villus sampling
FF	fetal fraction
FN	false negative
FP	false positive
FQA	fetal quantity assay
GC%	the proportion of G and C bases to all bases
IG	immunoglobulin
IVF	in vitro fertilization
MA	maternal age
MAF	minor allele frequency
MALDI-TOF MS	matrix-assisted laser desorption/ionization time-of-flight mass spectrometry
MW	maternal weight
NATUS	Next-Generation Aneuploidy Test Using SNPs
NGS	next-generation sequencing
NIPT	non-invasive prenatal testing
NPC	non-pregnant control
PAPP-A	pregnancy-associated plasma protein-A
PAR	pseudo-autosomal region
PGT	preimplantation genetic testing
RT-PCR	real-time poly chain reaction
SCA	sex chromosome aneuploidy
SNP	single-nucleotide polymorphism
STR	short tandem repeat
T13/18/21	trisomy 13/18/21
TN	trye negative
TP	true positive
WG	whole genome
uCG	unmodified CG dinucleotide
VAF_m_	maternal variant allele fraction
VAF_p_	paternal variant allele fraction

## References

[1] Elchalal U., Shachar I.B., Peleg D., Schenker J.G. (2004). Maternal mortality following diagnostic 2nd-trimester amniocentesis. Fetal Diagn. Ther..

[2] Seeds J.W. (2004). Diagnostic mid trimester amniocentesis: How safe?. Am. J. Obstet. Gynecol..

[3] Samura O., Okamoto A. (2020). Causes of aberrant non-invasive prenatal testing for aneuploidy: A systematic review. Taiwan. J. Obstet. Gynecol..

[4] Lo Y.M., Corbetta N., Chamberlain P.F., Rai V., Sargent I.L., Redman C.W., Wainscoat J.S. (1997). Presence of fetal DNA in maternal plasma and serum. Lancet.

[5] Harraway J. (2017). Non-invasive prenatal testing. Aust. Fam. Physician.

[6] Palomaki G.E., Chiu R., Pertile M.D., Sistermans E.A., Yaron Y., Vermeesch J.R., Vora N.L., Best R.G., Wilkins-Haug L. (2020). International Society for Prenatal Diagnosis Position Statement: Cell free (cf)DNA screening for Down syndrome in multiple pregnancies. Prenat. Diagn..

[7] Chiu R.W.K., Akolekar R., Zheng Y.W.L., Leung T.Y., Sun H., Chan K.C.A., Lun F.M.F., Go A.T.J.I., Lau E.T., To W.W.K. (2011). Non-invasive prenatal assessment of trisomy 21 by multiplexed maternal plasma DNA sequencing: Large scale validity study. BMJ.

[8] Pantiukh K.S., Chekanov N.N., Zaigrin I.V., Zotov A.M., Mazur A.M., Prokhortchouk E.B. (2016). Report on noninvasive prenatal testing: Classical and alternative approaches. F1000Research.

[9] Canick J.A., Palomaki G.E., Kloza E.M., Lambert-Messerlian G.M., Haddow J.E. (2013). The impact of maternal plasma DNA fetal fraction on next generation sequencing tests for common fetal aneuploidies. Prenat. Diagn..

[10] Ioannides M., Achilleos A., Kyriakou S., Kypri E., Loizides C., Tsangaras K., Constantinou L., Koumbaris G., Patsalis P.C. (2020). Development of a new methylation-based fetal fraction estimation assay using multiplex ddPCR. Mol. Genet. Genom. Med..

[11] Sparks A.B., Struble C.A., Wang E.T., Song K., Oliphant A. (2012). Noninvasive prenatal detection and selective analysis of cell-free DNA obtained from maternal blood: Evaluation for trisomy 21 and trisomy 18. Am. J. Obstet. Gynecol..

[12] Norton M.E., Jacobsson B., Swamy G.K., Laurent L.C., Ranzini A.C., Brar H., Tomlinson M.W., Pereira L., Spitz J.L., Hollemon D. (2015). Cell-free DNA analysis for noninvasive examination of trisomy. N. Engl. J. Med..

[13] Dar P., Curnow K.J., Gross S.J., Hall M.P., Stosic M., Demko Z., Zimmermann B., Hill M., Sigurjonsson S., Ryan A. (2014). Clinical experience and follow-up with large scale single-nucleotide polymorphism-based noninvasive prenatal aneuploidy testing. Am. J. Obstet. Gynecol..

[14] Porreco R.P., Garite T.J., Maurel K., Marusiak B., Ehrich M., van den Boom D., Deciu C., Bombard A., Obstetrix Collaborative Research Network (2014). Noninvasive prenatal screening for fetal trisomies 21, 18, 13 and the common sex chromosome aneuploidies from maternal blood using massively parallel genomic sequencing of DNA. Am. J. Obstet. Gynecol..

[15] Martin J.A., Hamilton B.E., Osterman M.J. (2012). Three decades of twin births in the United States, 1980–2009. NCHS Data Brief.

[16] Mansour R., Serour G., Aboulghar M., Kamal O., Al-Inany H. (2010). The impact of vanishing fetuses on the outcome of ICSI pregnancies. Fertil. Steril..

[17] Curnow K.J., Wilkins-Haug L., Ryan A., Kırkızlar E., Stosic M., Hall M.P., Sigurjonsson S., Demko Z., Rabinowitz M., Gross S.J. (2015). Detection of triploid, molar, and vanishing twin pregnancies by a single-nucleotide polymorphism-based noninvasive prenatal test. Am. J. Obstet. Gynecol..

[18] Kimelman D., Pavone M.E. (2021). Non-invasive prenatal testing in the context of IVF and PGT-A. Best practice & research. Clin. Obstet. Gynaecol..

[19] Revello R., Sarno L., Ispas A., Akolekar R., Nicolaides K.H. (2016). Screening for trisomies by cell-free DNA testing of maternal blood: Consequences of a failed result. Ultrasound Obstet. Gynecol..

[20] Scott F.P., Menezes M., Palma-Dias R., Nisbet D., Schluter P., da Silva Costa F., McLennan A.C. (2018). Factors affecting cell-free DNA fetal fraction and the consequences for test accuracy. J. Matern. Fetal Neonatal Med..

[21] Lee M.S., Cantonwine D., Little S.E., McElrath T.F., Parry S.I., Lim K.H., Wilkins-Haug L.E. (2015). Angiogenic markers in pregnancies conceived through in vitro fertilization. Am. J. Obstet. Gynecol..

[22] Amor D.J., Xu J.X., Halliday J.L., Francis I., Healy D.L., Breheny S., Baker H.W., Jaques A.M. (2009). Pregnancies conceived using assisted reproductive technologies (ART) have low levels of pregnancy-associated plasma protein-A (PAPP-A) leading to a high rate of false-positive results in first trimester screening for Down syndrome. Hum. Reprod..

[23] Lee T.J., Rolnik D.L., Menezes M.A., McLennan A.C., da Silva Costa F. (2018). Cell-free fetal DNA testing in singleton IVF conceptions. Hum. Reprod..

[24] Lambert-Messerlian G., Dugoff L., Vidaver J., Canick J.A., Malone F.D., Ball R.H., Comstock C.H., Nyberg D.A., Saade G., Eddleman K. (2006). First- and second-trimester Down syndrome screening markers in pregnancies achieved through assisted reproductive technologies (ART): A FASTER trial study. Prenat. Diagn..

[25] Futch T., Spinosa J., Bhatt S., de Feo E., Rava R.P., Sehnert A.J. (2013). Initial clinical laboratory experience in noninvasive prenatal testing for fetal aneuploidy from maternal plasma DNA samples. Prenat. Diagn..

[26] Bianchi D.W., Chiu R.W.K. (2018). Sequencing of circulating cell-free DNA during pregnancy. N. Engl. J. Med..

[27] Dharajiya N.G., Grosu D.S., Farkas D.H., McCullough R.M., Almasri E., Sun Y., Kim S.K., Jensen T., Salvidar S.-J., Topol E.J. (2018). Incidental detection of maternal neoplasia in noninvasive prenatal testing. Clin. Chem..

[28] Cohen P.A., Flowers N., Tong S., Hannan N., Pertile M.D., Hui L. (2016). Abnormal plasma DNA profiles in early ovarian cancer using a non-invasive prenatal testing platform: Implications for cancer screening. BMC Med..

[29] Ji X., Chen F., Zhou Y., Li J., Yuan Y., Mo Y., Liu Q., Tseng J.Y., Shih-Chieh Lin D., Shen S.H. (2018). Copy number variation profile in noninvasive prenatal testing (NIPT) can identify co-existing maternal malignancies: Case reports and a literature review. Taiwan. J. Obstet. Gynecol..

[30] Pavlidis N.A. (2002). Coexistence of pregnancy and malignancy. Oncologist.

[31] Bianchi D.W., Chudova D., Sehnert A.J., Bhatt S., Murray K., Prosen T.L., Garber J.E., Wilkins-Haug L., Vora N.L., Warsof S. (2015). Noninvasive Prenatal Testing and Incidental Detection of Occult Maternal Malignancies. JAMA.

[32] Bianchi D.W., Parsa S., Bhatt S., Halks-Miller M., Kurtzman K., Sehnert A.J., Amy S. (2015). Fetal sex chromosome testing by maternal plasma DNA sequencing: Clinical laboratory experience and biology. Obstet. Gynecol..

[33] Gregg A.R., Skotko B.G., Benkendorf J.L., Monaghan K.G., Bajaj K., Best R.G., Klugman S., Watson M.S., on behalf of the ACMG Noninvasive Prenatal Screening Work Group (2016). Noninvasive prenatal screening for fetal aneuploidy, 2016 update: A position statement of the American College of Medical Genetics and Genomics. Genet. Med..

[34] Wang E., Batey A., Struble C., Musci T., Song K., Oliphant A. (2013). Gestational age and maternal weight effects on fetal cell-free DNA in maternal plasma. Prenat. Diagn..

[35] Wald N.J., Lau K.W., Bestwick J.P., Old R.W., Huttly W.J., Cheng R. (2018). Specifying a Gold Standard for the Validation of Fetal Fraction Estimation in Prenatal Screening. Clin. Chem..

[36] Peng X.L., Jiang P. (2017). Bioinformatics Approaches for Fetal DNA Fraction Estimation in Noninvasive Prenatal Testing. Int. J. Mol. Sci..

[37] Lun F.M., Chiu R.W.K., Sun K., Leung T.Y., Jiang P., Chan K.C.A., Sun H., Lo Y.M.D. (2013). Noninvasive prenatal methylomic analysis by genomewide bisulfite sequencing of maternal plasma DNA. Clin. Chem..

[38] Lister R., Pelizzola M., Dowen R.H., Hawkins R.D., Hon G., Tonti-Filippini J., Nery J.R., Lee L., Ye Z., Ngo Q.-M. (2009). Human DNA methylomes at base resolution show widespread epigenomic differences. Nature.

[39] Lim J.H., Lee D.E., Park S.Y., Kim D.J., Ahn H.K., Han Y.J., Kim M.Y., Ryu H.M. (2014). Disease specific characteristics of fetal epigenetic markers for non-invasive prenatal testing of trisomy 21. BMC Med. Genom..

[40] Poon L.L., Leung T.N., Lau T.K., Chow K.C., Lo Y.M. (2002). Differential DNA methylation between fetus and mother as a strategy for detecting fetal DNA in maternal plasma. Clin. Chem..

[41] Chim S.S., Tong Y.K., Chiu R.W., Lau T.K., Leung T.N., Chan L.Y.S., Oudejans C.B.M., Ding C., Lo Y.M.D. (2005). Detection of the placental epigenetic signature of the maspin gene in maternal plasma. Proc. Natl. Acad. Sci. USA.

[42] Chan K.C., Ding C., Gerovassili A., Yeung S.W., Chiu R.W., Leung T.N., Lau T.K., Chim S.S., Chung G.T., Nicolaides K.H. (2006). Hypermethylated RASSF1A in maternal plasma: A universal fetal DNA marker that improves the reliability of noninvasive prenatal diagnosis. Clin. Chem..

[43] Staševskij Z., Gibas P., Gordevičius J., Kriukienė E., Klimašauskas S. (2017). Tethered oligonucleotide-primed sequencing, TOP-Seq: A high-resolution economical approach for DNA epigenome profling. Mol. Cell.

[44] Gibas P., Narmontė M., Staševskij Z., Gordevičius J., Klimašauskas S., Kriukienė E. (2020). Precise genomic mapping of 5-hydroxymethylcytosine via covalent tether-directed sequencing. PLoS Biol..

[45] Gordevičius J., Narmontė M., Gibas P., Kvederavičiūtė K., Tomkutė V., Paluoja P., Krjutškov K., Salumets A., Kriukienė E. (2020). Identification of fetal unmodified and 5-hydroxymethylated CG sites in maternal cell-free DNA for non-invasive prenatal testing. Clin. Epigenet..

[46] Nygren A.O., Dean J., Jensen T.J., Kruse S., Kwong W., van den Boom D., Ehrich M. (2010). Quantification of fetal DNA by use of methylation-based DNA discrimination. Clin. Chem..

[47] Sims D., Sudbery I., Ilot N.E., Heger A., Ponting C.P. (2014). Sequencing depth and coverage: Key considerations in genomic analyses. Nat. Rev. Genet..

[48] Jiang P., Peng X., Su X., Sun K., Yu S.C.Y., Chu W.I., Leung T.Y., Sun H., Chiu R.W.K., Lo Y.M.D. (2016). FetalQuant^SD^: Accurate quantification of fetal DNA fraction by shallow-depth sequencing of maternal plasma DNA. NPJ Genom. Med..

[49] Phillips C. (2009). SNP databases. Methods Mol. Biol..

[50] Kim M., Kim J.H., Kim K., Kim S. (2018). Cost-effective and accurate method of measuring fetal fraction using SNP imputation. Bioinformatics.

[51] Das S., Forer L., Schönherr S., Sidore C., Locke A.E., Kwong A., Vrieze S.I., Chew E.Y., Levy S., McGue M. (2016). Next-generation genotype imputation service and method. Nat. Genet..

[52] Lee S., Abecasis G.R., Boehnke M., Lin X. (2014). Rare-variant association analysis: Study designs and statistical tests. Am. J. Hum. Genet..

[53] McCarthy S., Das S., Kretzschmar W., Delaneau O., Wood A.R., Teumer A., Kang H.M. (2016). A reference panel of 64, 976 haplotypes for genotype imputation. Nat. Genet..

[54] Zhang J., Li J., Saucier J.B., Feng Y., Jiang Y., Sinson J., McCombs A.K., Schmitt E.S., Peacock S., Chen S. (2019). Non-invasive prenatal sequencing for multiple Mendelian monogenic disorders using circulating cell-free fetal DNA. Nat. Med..

[55] Kim S.K., Hannum G., Geis J., Tynan J., Hogg G., Zhao C., Jensen T.J., Mazloom A.R., Oeth P., Ehrich M. (2015). Determination of fetal DNA fraction from the plasma of pregnant women using sequence read counts. Prenat. Diagn..

[56] Jensen T.J., Zwiefelhofer T., Tim R.C., Džakula Ž., Kim S.K., Mazloom A.R., Zhu Z., Tynan J., Lu T., McLennan G. (2013). High-throughput massively parallel sequencing for fetal aneuploidy detection from maternal plasma. PLoS ONE.

[57] Yu S.C., Chan K.C., Zheng Y.W., Jiang P., Liao G.J., Sun H., Akolekar R., Leung T.Y., Go A.T., van Vugt J.M. (2014). Size-based molecular diagnostics using plasma DNA for noninvasive prenatal testing. Proc. Natl. Acad. Sci. USA.

[58] Jiang C., Pugh B.F. (2009). Nucleosome positioning and gene regulation: Advances through genomics. Nat. Rev. Genet..

[59] Dheedene A., Sante T., De Smet M., Vanbellinghen J.F., Grisart B., Vergult S., Janssens S., Menten B. (2016). Implementation of non-invasive prenatal testing by semiconductor sequencing in a genetic laboratory. Prenat. Diagn..

[60] Bedon L., Vuch J., Monego S.D., Meroni G., Pecile V., Licastro D. (2021). An online tool for fetal fraction prediction based on direct size distribution analysis of maternal cell-free DNA. BioTechniques.

[61] Koenig M., Moisan J.P., Heilig R., Mandel J.L. (1985). Homologies between X and Y chromosomes detected by DNA probes: Localisation and evolution. Nucleic Acids Res..

[62] Mangs A.H., Morris B.J. (2007). The Human Pseudoautosomal Region (PAR): Origin, Function and Future. Curr. Genom..

[63] Mazloom A.R., Džakula Ž., Oeth P., Wang H., Jensen T., Tynan J., McCullough R., Saldivar J.S., Ehrich M., van den Boom D. (2013). Noninvasive prenatal detection of sex chromosomal aneuploidies by sequencing circulating cell-free DNA from maternal plasma. Prenat. Diagn..

[64] Hudecova I., Sahota D., Heung M.M., Jin Y., Lee W.S., Leung T.Y., Lo Y.M., Chiu R.W. (2014). Maternal plasma fetal DNA fractions in pregnancies with low and high risks for fetal chromosomal aneuploidies. PLoS ONE.

[65] Gazdarica J., Hekel R., Budis J., Kucharik M., Duris F., Radvanszky J., Turna J., Szemes T. (2019). Combination of Fetal Fraction Estimators Based on Fragment Lengths and Fragment Counts in Non-Invasive Prenatal Testing. Int. J. Mol. Sci..

[66] Samango-Sprouse C., Banjevic M., Ryan A., Sigurjonsson S., Zimmermann B., Hill M., Hall M.P., Westemeyer M., Saucier J., Demko Z. (2013). SNP-based non-invasive prenatal testing detects sex chromosome aneuploidies with high accuracy. Prenat. Diagn..

[67] Van Beek D.M., Straver R., Weiss M.M., Boon E., Huijsdens-van Amsterdam K., Oudejans C., Reinders M., Sistermans E.A. (2017). Comparing methods for fetal fraction determination and quality control of NIPT samples. Prenat. Diagn..

[68] Bayindir B., Dehaspe L., Brison N., Brady P., Ardui S., Kammoun M., van der Veken L. (2015). Noninvasive prenatal testing using a novel analysis pipeline to screen for all autosomal fetal aneuploidies improves pregnancy management. Eur. J. Hum. Genet..

[69] Straver R., Oudejans C.B., Sistermans E.A., Reinders M.J. (2016). Calculating the fetal fraction for noninvasive prenatal testing based on genome-wide nucleosome profiles. Prenat. Diagn..

[70] Hestand M.S., Bessem M., van Rijn P., de Menezes R.X., Sie D., Bakker I., Boon E., Sistermans E.A., Weiss M.M. (2019). Fetal fraction evaluation in non-invasive prenatal screening (NIPS). Eur. J. Hum. Genet. EJHG.

[71] Miceikaitė I., Brasch-Andersen C., Fagerberg C., Larsen M.J. (2021). Total number of reads affects the accuracy of fetal fraction estimates in NIPT. Mol. Genet. Genom. Med..

[72] Tam J., Chan Y.M., Tsang S.Y., Yau C.I., Yeung S.Y., Au K.K., Chow C.K. (2020). Noninvasive prenatal paternity testing by means of SNP-based targeted sequencing. Prenat. Diagn..

[73] Amant F., Verheecke M., Wlodarska I., Dehaspe L., Brady P., Brison N., Van Den Bogaert K., Dierickx D., Vandecaveye V., Tousseyn T. (2015). Presymptomatic Identification of Cancers in Pregnant Women During Noninvasive Prenatal Testing. JAMA Oncol..

[74] Chan K.C.A., Leung S.F., Yeung S.W., Chan A.T., Lo Y.M.D. (2008). Persistent aberrations in circulating DNA integrity after radiotherapy are associated with poor prognosis in nasopharyngeal carcinoma patients. Clin. Cancer Res..

[75] Scheijen B., Meijers R., Rijntjes J., van der Klift M.Y., Möbs M., Steinhilber J., Reigl T., van den Brand M., Kotrová M., Ritter J.M. (2019). Next-generation sequencing of immunoglobulin gene rearrangements for clonality assessment: A technical feasibility study by EuroClonality-NGS. Leukemia.

[76] Zheng Y.W., Chan K.C., Sun H., Jiang P., Su X., Chen E.Z., Lun F.M., Hung E.C., Lee V., Wong J. (2012). Nonhematopoietically derived DNA is shorter than hematopoietically derived DNA in plasma: A transplantation model. Clin. Chem..

[77] Kaposy C. (2013). A disability critique of the new prenatal test for down syndrome. Kennedy Inst. Ethics J..

[78] How B., Smidt A., Wilson N.J., Barton R., Valentin C. (2019). ‘We would have missed out so much had we terminated’: What fathers of a child with Down syndrome think about current non-invasive prenatal testing for Down syndrome. J. Intellect. Disabil..

[79] Berry S. “Study: 96% of 5,577 Biologists Affirm Human Life Begins at Fertilization”, Breitbart, July 11, 2019. breitbart.com/politics/2019/07/11/study-96-of-5577-biologists-affirm-human-life-begins-at-fertilization.

[80] Bunnik E.M., Kater-Kuipers A., Galjaard R.H., de Beaufort I. (2020). Why NIPT should be publicly funded. J. Med. Ethics.

